# Delayed hospitalization increases mortality in displaced femoral neck fracture patients

**DOI:** 10.3109/17453670903506666

**Published:** 2009-12-04

**Authors:** Arunas Vertelis, Otto Robertsson, Sarunas Tarasevicius, Hans Wingstrand

**Affiliations:** ^1^Department of Orthopedics, Kaunas Medical University, Kaunas, Lithuania; ^2^Department of Orthopedics Lund University and Lund University Hospital, Lund, Sweden

## Abstract

**Background and purpose** Reports regarding the relationship between delayed surgery and mortality in femoral neck fracture patients are contradictory. We could not find any study in the literature investigating delayed arrival to hospital and delayed surgery as separate factors affecting mortality in femoral neck fracture patients, which was the purpose of our study.

**Patients and methods** We analyzed 265 consecutive patients with displaced femoral neck fractures. We recorded the time period from trauma to admission, and to surgery, and correlated it to mortality during the first postoperative year.

**Results** We found that arrival within 6 hours had 0.4 times (CI 0.2–0.8) reduction of the risk of death within 1 year compared to those who arrived later, whereas delayed surgery after admission did not have a statistically significant effect on mortality.

**Interpretation** Femoral neck fracture patients who arrived at hospital 6 hours or later after the trauma had increased mortality.

## Introduction

It has been shown that elderly patients who sustain femoral neck fractures have increased mortality compared to the general population (Zuckerman 1996, Roberts and Goldacre 2003). Many parameters such as the type of surgery, the preoperative condition, and age may affect mortality (Blomfeldt et al. 2007, Hommel et al. 2008). Delayed surgery has been suggested to increase mortality (Bottle and Aylin 2006, Sebestyén et al. 2008), although there is no strong evidence to support this. A few studies have analyzed delayed surgery, after admission, as a separate factor affecting mortality. In a retrospective study, Bottle et al. (2006) investigated 129,522 admissions in 151 hospitals and found that 1 day of delay before surgery was associated with increased mortality. Sebestyen et al. (2008) analyzed 3,783 hip fractures and found that delay of surgery for more than 12 hours was associated with increased mortality. In an observational study involving 2,660 patients, Moran et al. (2005) found that delay of surgery for 4 days or more was associated with increased mortality at 30 days, 90 days, and 1 year after surgery. However, in a retrospective study of 3,981 patients, Majumdar et al. (2006) found no correlation between timing of surgery and mortality. All these studies included mixed patient groups, i.e. different types of fractures and surgical procedures. None analyzed delay in arrival at hospital as a separate factor possibly affecting outcome.

Orosz et al. (2002) investigated the intervals between trauma and hospitalization in femoral neck fractures and analyzed the reasons for delay. They found that out of the 571 patients, 99 (17%) had arrived at the hospital more than 24 hours after the trauma.

As we are not aware of studies specifically evaluating the relation between mortality and delayed arrival/surgery, we investigated whether time from fracture to hospital admission and/or time from admission to surgery had any effect on the 1 year mortality in patients with displaced femoral neck fracture.

## Patients and methods

Between January 1, 2003 and December 31, 2005, 267 patients with fresh femoral neck fractures were admitted to Kaunas Red Cross Hospital for surgery. 61 had total hip arthroplasty (THA) because of signs of osteoarthritis in the fractured hip or comminuted fractures involving the femoral head, 204 were treated with osteosynthesis, and 2 patients had to be excluded because of missing data. 4 patients with femoral neck fractures classified as Garden I or II were treated nonoperatively and were also excluded from the study. The remaining femoral neck fractures included in the study were classified as Garden III or IV, which was determined from anterior-posterior radiographs. We have no clear explanation for the low number of Garden I and II fractures. However, one reason could be that patients with non-displaced fractures delayed seeking medical attention due to having less pain than patients with displaced fractures. Subsequently, after the pain had continued or increased, they were then admitted with an already displaced femoral neck fracture.

Almost all patients had been able to walk independently before the trauma.

The institution in Lithuania where the study was conducted is located in a city with 300,000 inhabitants and it also accepts patients from the suburban area within a 50-km radius. It is estimated that 2 hours at most will pass from the health services receiving information about the trauma and the patient's arrival at the hospital.

After arrival at the emergency room, the patient was asked about the time of fracture and the answer was then confirmed by relatives or any other person accompanying the patient. The time of admission and the time when the reduction of the femoral neck fracture was performed in the operating room was recorded. Immediately after admission, blood samples for hemoglobin, potassium, and urea tests were taken.

The American Society of Anaesthesiologists classification of physical status (ASA score) was recorded by an anesthesiologist directly after admission.

Spinal anesthesia was used in all patients. In osteosynthesis patients, closed reduction was performed under biplanar fluoroscopic control and the fracture was fixed with the 3 Ullevaal hip screws (Orthovita, Norway) (Lykke et al. 2003). The THA patients were operated using a posterior approach and both components were cemented.

All patients received the same infection prophylaxis with cefuroxim, and thrombosis prophylaxis with low-molecular-weight heparin. For osteosynthesis patients, partial weight bearing was allowed on the first day, and full weight bearing was allowed 6 weeks postoperatively. The THA patients were mobilized on the day after surgery. Otherwise, the same rehabilitation program was used.

Information on whether or not patients were alive 1 year after surgery was gathered from the National Patients' Fund register, and for deceased patients the cause of death was also noted.

When analyzing the time from fracture to arrival, the median value was 6 hours and we divided the patients into two groups according to whether they had arrived at hospital within 6 hours or later than 6 hours. Similarly, we divided the patients into two groups according to whether they had undergone surgery within the median waiting time of 7 hours, or later.

The study was approved by the local ethics committee (BE-2-12).

### Statistics

In Cox regression, the two groups of patients who arrived and were operated early and late, respectively, were used as categorical variables, as was the sex and type of operation. Arrival within 6 hours was compared to arrival later. Surgery within 7 hours of arrival was compared to surgery later, and males were used as a reference for the females. Age was used as continuous variable and the risk expresses the change in risk for every year in increased age. The ASA grade and the blood test values were not included in the Cox regression analysis, due to possible inter-correlations between these variables and also due to possible inter-correlations with age and time to arrival or surgery. Independent samples t-test was used to compare the means of numerical variables. Chi-squared test was used to compare the proportions between categorical variables. A p-value of < 0.05 was considered significant. SPSS software was used for the calculations.

## Results

There were 179 women and 86 men, with a mean age of 77 (SD 9) years in women and 72 (SD 14) years in men. Osteosynthesis was performed in 204 patients and THA in 61 patients.

The mean time from trauma to arrival at the hospital was 50 (SD 122) h while the median was 6 hours. The mean time from admission to surgery was 18 (SD 40) h for osteosynthesis; for THA patients, it was 114 (SD 122) h (p = 0.001). The total mean time from admission to surgery was 40 (SD 80) h and the median time was 7 h.

None of the patients died before surgery. 38 patients died (14%, 23 of whom were women) during the first year after surgery. 12 patients died in the group of patients who arrived earlier than 6 hours after the trauma and 26 patients died in the late arrivals group. 19 patients died in the group of patients who were operated before 7 hours, and 19 patients died in the group operated later. In all deceased cases, the causes of death had been recorded as cardiovascular.

During the first postoperative year, 6 osteosyntheses had to be converted to THA while 7 patients had their pins removed. All of these 13 patients were alive at the end of the 1-year follow-up and were not excluded.

No patients had delayed seeking medical attention because of difficulties in contacting relatives or caregivers. In a few cases, the delay in arrival at hospital was caused by the fact that the fracture had been misdiagnosed by the general family doctor. The remaining patients did not consider the trauma to be important and delayed seeking medical attention.

Patients who arrived within 6 hours of fracture had higher hemoglobin, while their urea was lower than in patients who arrived later. Potassium level, age, ASA grade, and sex were similar in the 2 groups (Table 1). Cox regression analysis showed that the group that arrived within 6 hours after the fracture had a lower mortality than the group that arrived later. As expected, age had an effect on mortality. There was a tendency for females to have lower mortality than males, although the difference was not significant (p = 0.06). The mortality rate was similar in patients who had surgery within 7 hours of arrival or later, and whether an osteosynthesis or a THA had been performed (Table 2).

**Table 1.** Means and t-test results of hemoglobin values, potassium values, urea values, age, and ASA grades, comparing patients who arrived at hospital before or later than 6 hours after fracture

Variables	Admitted before 6 hours (n = 132)	Admitted later than 6 hours (n = 133)	p-value
Age, years (SD)	75 (11)	76 (11)	0.5
ASA grade (SD)	2.3 (0.6)	2.4 (0.6	0.1
Hemoglobin, g/L (SD)	131 (17)	124 (21)	0.004
Potassium, mg/L (SD)	4.2 (0.5)	4.2 (0.5)	0.9
Urea, mg/L (SD)	7.0 (2.4)	8.2 (3.5)	0.002

**Table 2.** Relative risk of death within 1 year. Cox regression analysis

	RR	CI	p-value
Women (reference: men)	0.5	0.3–1.0	0.06
Age (change in risk for each year in increased age)	1.1	1.0–1.1	< 0.001
Arrival within 6 hours (reference: arrival later)	0.4	0.2–0.8	0.01
Surgery within 7 hours of arrival (reference: surgery later)	0.7	0.4–1.5	0.9
Osteosynthesis (reference: THA)	1.4	0.6–3	0.4

RR: relative risk; CI: 95% confidence interval.

## Discussion

We found that the mortality rate during the first year after surgery was doubled in patients who had arrived later than 6 hours after the trauma. This might be related to delay in medical treatment, such as fluid restitution and thrombotic prophylaxis. There have been previous reports about a correlation between delayed surgery and mortality (Bottle and Aylin 2006, Sebestyén et al. 2008). However, the delay in surgery analyzed was that between admission and surgery, and it did not take delayed arrival at hospital into account. We are not aware of any previous studies that have examined delayed arrival alone in femoral neck fracture patients. Vidal et al. (2009) retrospectively investigated the effect of delay in arrival and delay in surgery on mortality in the one hospital. That study included all types of hip fractures and they found that neither time to admission nor time between admission and surgery affected mortality. However, in that study no separate analysis of femoral neck fractures was performed; nor were patients followed up to 1 year after surgery, as in the present study. Our results indicate that it is delayed arrival at hospital that is of importance, not delayed surgery after arrival.

The correlation we found between late arrival and lower hemoglobin level and higher urea is understandable in the light of blood loss and dehydration. It is of relevance to our study that the ASA levels in the two groups, and thus the general health of both sets of patients, were similar.

When comparing the patients who were operated within 7 hours of admission and those operated later, we found no difference in mortality. Delayed surgery is usually caused by the patient's need for medical attention preoperatively or to logistic problems, especially if waiting for arthroplasty. The latter is reflected by the fact that the THA patients had to wait considerably longer for surgery than those waiting for osteosynthesis. Even though surgery in some patients was delayed for medical reasons, we did not find that this delay affected mortality. This appears to indicate that the medical attention they received improved their prognosis sufficiently for the mortality rate not to be affected in a negative way.

The effect of type of surgery on mortality in femoral neck fractures has been discussed. Davison et al. (2001) reported lower mortality in patients treated with osteosynthesis as compared to THA. However, more recent studies comparing internal fixation and arthroplasty have found no difference in mortality between the methods (Parker et al. 2002, Tidermark et al. 2003), which is what we found.

14% of the patients died within 1 year. The reported 1 year mortality after femoral neck fractures varies from 14% to 36% (Rogmark et al. 2002, Blomfeldt et al. 2005, Miyamoto et al. 2008). Thus, we assume that our patients were similar to others regarding their general health.

We conclude from this study that femoral neck fracture patients who arrived at hospital later than 6 hours after the trauma had increased mortality.
